# The Preventive and Therapeutic Effects of Intravenous Human Adipose-Derived Stem Cells in Alzheimer’s Disease Mice

**DOI:** 10.1371/journal.pone.0045757

**Published:** 2012-09-26

**Authors:** Saeromi Kim, Keun-A Chang, Jeong a. Kim, Hyeong-Geun Park, Jeong Chan Ra, Hye-Sun Kim, Yoo-Hun Suh

**Affiliations:** 1 Department of Pharmacology, College of Medicine, Neuroscience Research Institute, MRC, Seoul National University, Seoul, South Korea; 2 Stem Cell Research Center, RNL Bio Co., Ltd., Seoul, Republic of Korea; 3 Korea Brain Research Institute (KBRI), Daegu, South Korea; 4 Department of Pharmacology, Gachon University of Medicine and Science, Incheon, South Korea; Centre Hospitalier de l’Université Laval, Canada

## Abstract

Alzheimer’s disease (AD) is characterized by the accumulation of amyloid plaques and neurofibrillary tangles accompanied by cognitive dysfunction. The aim of the present study was to elucidate preventive and therapeutic potential of stem cells for AD. Among stem cells, autologous human adipose-derived stem cells (hASCs) elicit no immune rejection responses, tumorigenesis, or ethical problems. We found that intravenously transplanted hASCs passed through the BBB and migrated into the brain. The learning, memory and pathology in an AD mouse model (Tg2576) mice greatly improved for at least 4 months after intravenous injection of hASC. The number of amyloid plaques and Aβ levels decreased significantly in the brains of hASC-injected Tg mice compared to those of Tg-sham mice. Here, we first report that intravenously or intracerebrally transplanted hASCs significantly rescues memory deficit and neuropathology, in the brains of Tg mice by up-regulating IL-10 and VEGF and be a possible use for the prevention and treatment of AD.

## Introduction

AD is the most prevalent neurodegenerative disorder in The United States affecting approximately 5.3 million Americans [1]. AD is characterized by progressive loss in memory and as well as a decline in the ability to learn that is associated with neuronal death. Well known hallmarks of AD are neuritic plaques and neurofibrillary tangles [2], [3] and extensive inflammation [4]. Currently, no treatment has been developed to fully cure or prevent the progression of dementia that is associated with AD.

Therapeutic potentials of stem cells in several brain disorders are enticing researchers to apply stem cell-based therapies [5]–[7]. Neural stem cells have been shown to rescue memory impairment in AD model mice by releasing brain-derived neurotrophic factor (BDNF) [2]. Also, Bone Marrow-Derived Mesenchymal Stem Cells (BM-MSCs) alleviated Aβ deposition and memory deficits in AD model mice by modulating immune response [8]. However, it would almost be impossible to perform intravenous transplantation of neural stem cells and BM-MSCs.

Among stem cells, adipose-derived stem cells (ASCs), mesenchymal stem cells isolated from adipose tissue, are well known for their pluripotency and ability to differentiate into mesenchymal and non-mesenchymal lineages [9]. ASCs are readily accessible and show high proliferation rates in vitro with lower senescence ratios than BM-MSCs [10]. Considering clinical applications, ASCs are the most suitable source of stem cells due to the possibility of to intravenous transplantation of autologous ASCs with no immune rejections, ethical problems or tumorigenesis [11] and intravenous injection is the most convenient, simple and safest method. Therapeutic potential of intracerebral injection of human ASCs (hASCs) in neurodegenerative diseases was previously reported in Huntington’s disease (HD) and ischemia mouse models [12], [13]. However the pathogenesis of AD is very different from those of stroke and HD. Therefore these findings were not indicative if they would be beneficial in AD.

Here, we first confirmed that intravenously injected stem cells could enter the brain through BBB and hASCs could have beneficial effects in Tg2576, AD model mice by injecting hASCs in two ways: intra-venous and intra-cerebral injection. Intracerebral injection is intended to examine the therapeutic potential of hASCs in the early stage of the disease while intravenous injection is more related to preventing or delaying the onset of disease. With both injection methods, hASCs showed therapeutic or preventive potentials rescuing cognitive impairments and reducing Aβ pathology and especially, very simply, a convenient and safe intravenous injection of hASCs might be very useful for both the prevention and treatment of AD.

## Results

### Intravenous or Intracerebral hASC Transplantation Rescued Memory Impairments and Prominent Fluorescence Signals from hASCs were Detected in the Brains

**Figure 1:**
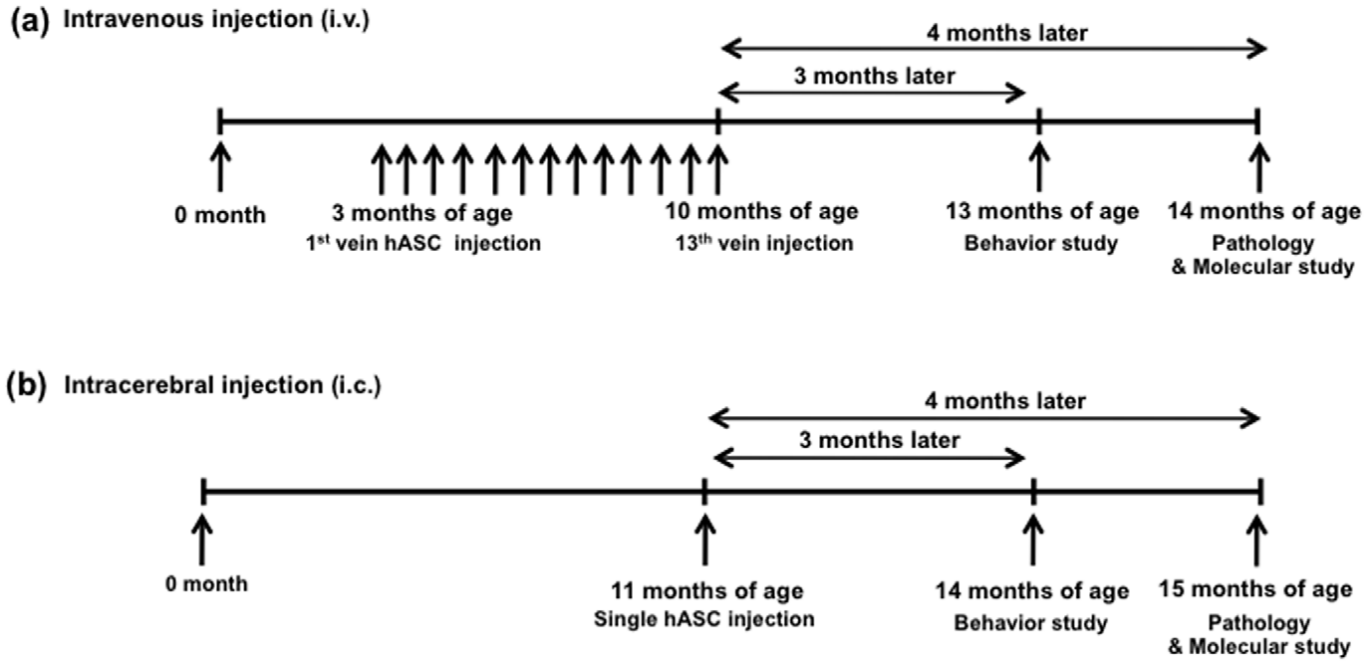
Experimental schemes of intravenous and intracerebral injections. (a) Experimental scheme of intravenous injection (i.v.). (b) Experimental scheme of intracerebral injection (i.c.).

The hASCs were intravenously transplanted into Tg2576 and WT mice biweekly a total of 13 times from 3 months of age (Fig. 1a) or bilaterally transplanted into the dentate gyrus (DG) of the hippocampus of the 11-month-old Tg2576 and age-matched wild type (WT) mice (Fig. 1b).

We checked whether intravenously transplanted hASCs passed through the BBB and migrated into the brain, we injected hASCs labeled with fluorescence magnetic nanoparticles into the tail vein of mice and monitored hASCs at 0, 1, 3 and 10 days after i.v. injection of labeled hASCs in live mice (Fig. S1a). One day after injection of labeled cells, the fluorescence signal was mostly detected in the liver, however some were detected in the brain of Tg2576 (Fig. S1a). On the 3^rd^ day after injection, prominent fluorescence signals from hASCs were detected in the brains, and we found that the cells remained in the brain up to 10 days (Fig. S1a). Fluorescence signals from the organs extracted 3 days after cell transplantation show that the cells had spread throughout the entire organs including the brain (Fig. S1b). After dissecting the brain into 5 distinct regions (olfactory bulb, hippocampus, cerebellum, brainstem, midbrain and cortex), we found fluorescent nanoparticle signals in all brain regions except the olfactory bulb (Fig. S1c). Our data clearly shows that the intravenously transplanted hASCs survive and migrate into the brain.

**Figure 2:**
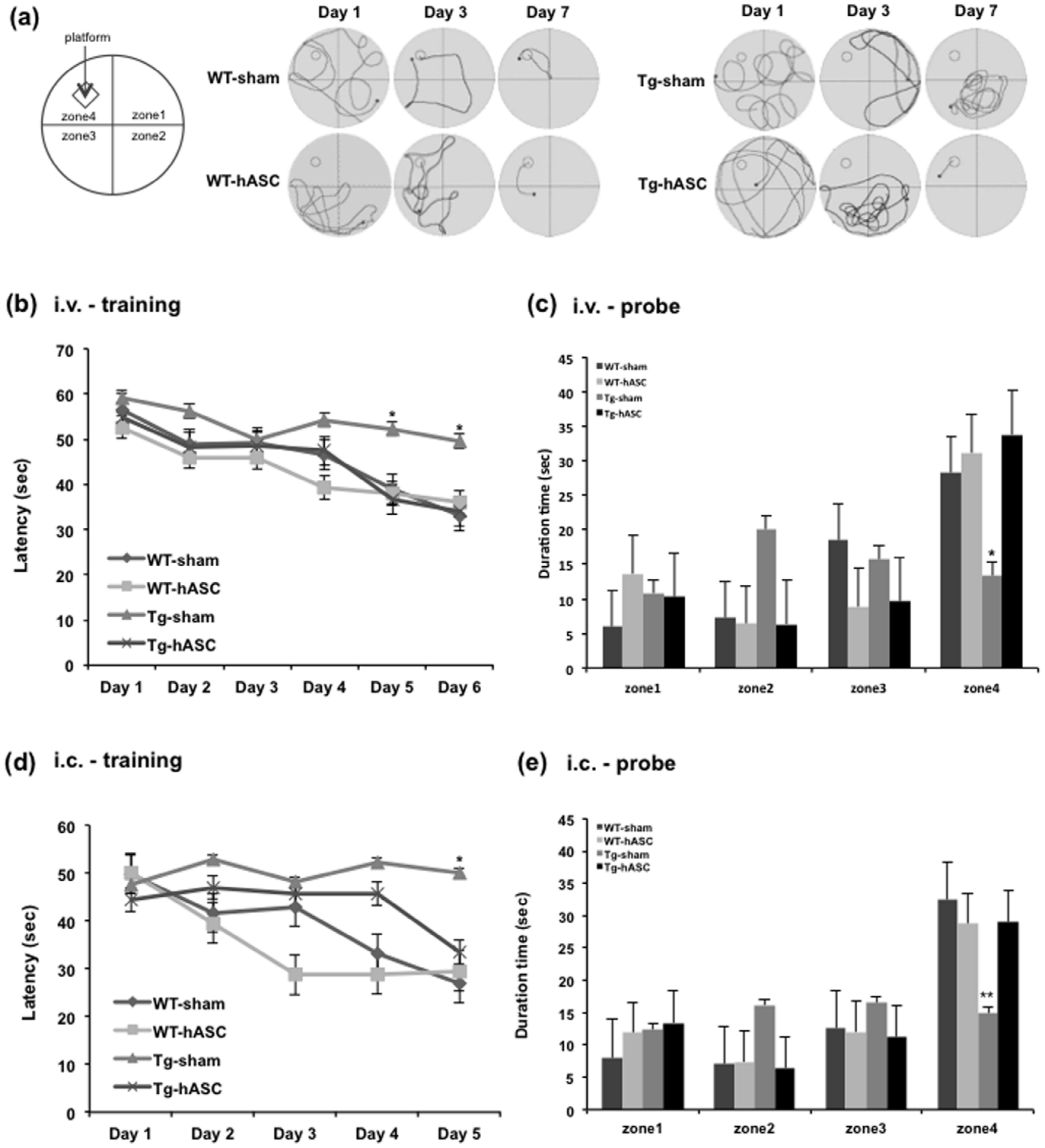
Intravenous and intracerebral injection of hASC attenuated learning and memory impairment in Tg2576 mouse brains. (a) A Morris water maze task was performed 3 months after final intravenous hASC injection. The path shapes of the movement of the mice during the training period were obtained. (b) The task was conducted for 7 consecutive days 4 months after the last intravenous injection. A significant difference was observed between the Tg-hASC group and the Tg-sham group from the 5^th^ day of the Morris water maze task. (c) The probe test was carried out 48 h after the final trial. The Tg-hASC group showed memory improvement compared to the Tg-sham group in zone 4 where the platform had been hidden (n = 11∼20 per group) (d) A Morris water maze task was performed 3 months after hASC i.c. injection. The task was conducted for 6 consecutive days. A significant difference was observed between the Tg-hASC group and the Tg-sham group on the 5^th^ day of the Morris water maze task. (e) The probe test was carried out 48 h after the final trial. The Tg-hASC group showed memory improvement compared to the Tg-sham group in zone 4 where the platform had been hidden (n = 10∼15 per group), All data are represented as mean ± SEM. Asterisk *, *P*<0.05, **, *P*<0.01 by one-way ANOVA.

To determine whether engrafted hASC transplantation improved cognitive deficits, we performed the Morris Water Maze 3 months after the final (13^th^) intravenous injection or single intracerebral hASC injection. With trainings repeating daily, WT-sham, WT-hASC and Tg-hASC groups found the hidden platform with less movement while the Tg-sham group kept wandering with no regular pattern (Fig. 2a). Analysis of the escape latency of each group showed significant difference between the Tg-hASC and Tg-sham groups (Fig. 2b and d). We found no noticeable difference between WT-sham and WT-hASC groups (Fig. 2b and d). 48 hours after the final trial, we performed the probe test without the platform and checked the duration of time spent in the zone 4 where the platform was previously hidden. The Tg-hASC group spent significantly more time in zone 4 than in other 3 zones (zones 1–3) (Fig. 2c and e), as WT groups did. However, in the case of Tg-sham, no significant difference between times spent in each zone was observed (Fig. 2c and e). These data show that both intravenous and intracerebral hASC transplantation improved spatial learning inTg2576 mice.

### Intravenous or Intracerebral hASC Transplantation Reduced the Number of Amyloid Plaques in the Brain

**Figure 3:**
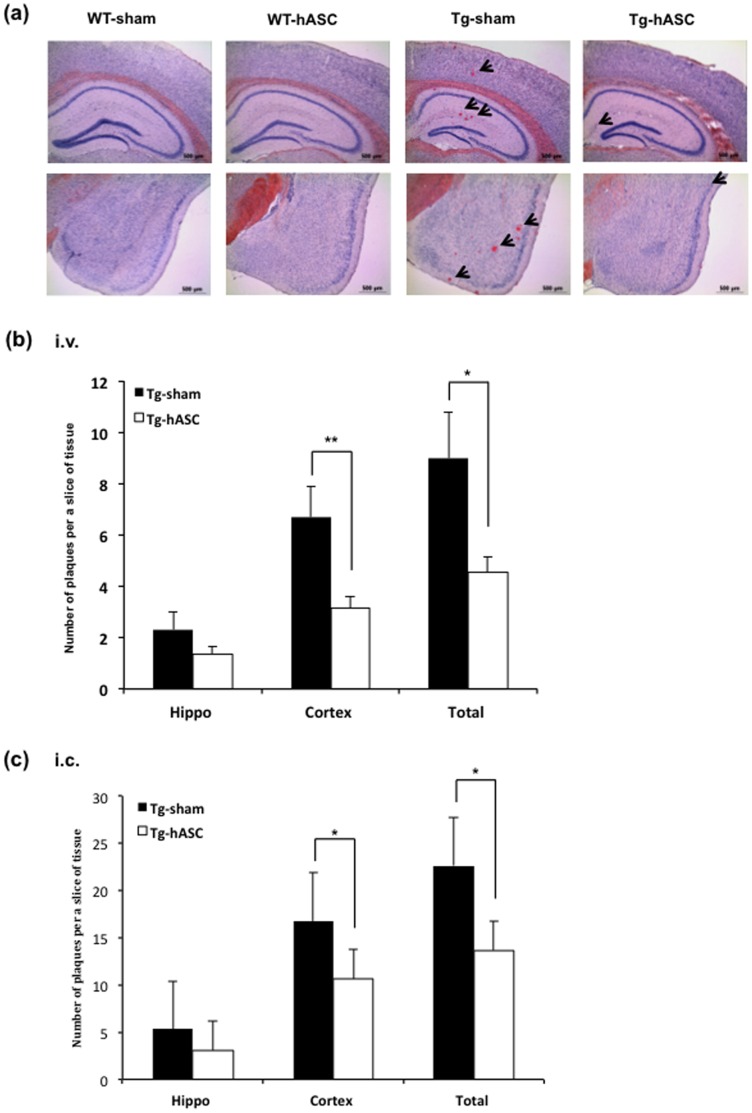
Intravenous and intracerebral injection of hASC reduced the number of amyloid plaques in Tg2576 mouse brains. (a) Congo red staining for the detection of amyloid plaques was carried out in the hippocampus of each group 4 months after (i.v.) injection. (b) 4 months after the 13^th^ (i.v.) injection, the number of plaques was counted in the hippocampal region of the Tg-hASC and the Tg-sham group. (c) At 4 months after hASC (i.c.) injection, the number of plaques was counted in the hippocampal region of Tg-hASC and Tg-sham groups. All data are represented as mean ± SEM (n = 9∼15 per group). Asterisk *, *P*<0.05, **, *P*<0.01 by one-way ANOVA.

To investigate whether hASC transplantation could alleviate toxic amyloid plaque formation, we performed Congo red staining on postmortem brains 4 months after injection with WT-sham, WT-hASC, Tg-sham and Tg-hASC mice. While Tg2576 mice showed amyloid plaque formation in almost all regions of the brain, there was no plaque observed in age-matched WT group mice (Fig. 3a).

Intravenously transplanted hASCs reduced the number of amyloid plaques in the 14-month-old Tg2576 mice brains (Fig. 3b). In the Tg-hASC group, there was a significant reduction in the cortex (from 6.67±1.21 to 3.17±0.43, p<0.01) and notable difference in the hippocampus (from 2.3127±0.71 to 1.38±0.28, p<0.14) compared to the Tg-sham group (Fig. 3b). There was a significant difference between intracerebrally transplanted hASC and sham groups of 15-month-old Tg2576 mice in both cortex (sham; 16.75±5.30, hASC; 9.11±2.78, p<0.05) and hippocampus (sham; 5.34±2.312, hASC; 2.29±1.082, p<0.05) (Fig. 3c).

These data suggest that the intravenous or intracerebral hASC transplantation improved the AD-like pathology of Tg2576 by reducing the number of plaques.

### Protein Levels of Aβ and APP-CT were Reduced after Intravenous or Intracerebral Injection of hASC

**Figure 4:**
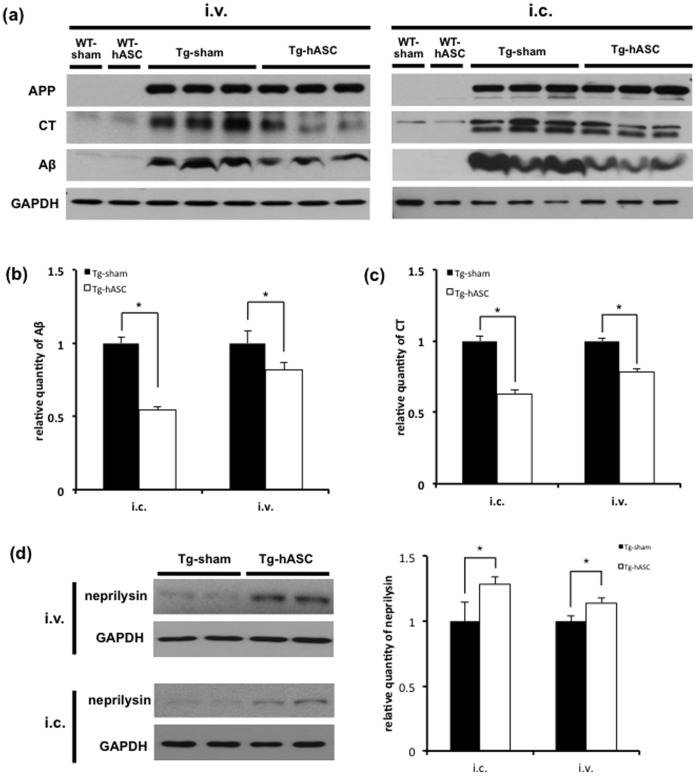
Intravenous and intracerebral injection of hASC reduced Aβ and APP-CT levels and increased neprilysin in Tg2576 mouse brains. (a) Western blot analysis was performed with lysates from the cortical region of the brains in each group using 6E10 and GAPDH antibodies 4 months after injection. (b, c) Aβ and CT expressions were normalized with those of APP and GAPDH for quantification (n = 5). (d) Neprilysin level was significantly increased in Tg-hASC group (n = 4). All data are represented as mean ± SEM. Asterisk *, *P*<0.05 by one-way ANOVA.

We examined protein levels of Amyloid Precursor Protein (APP), APP C-terminal fragment (APP-CT) and Aβ using 6E10 antibody 4 months after injection based on Congo red staining data (Fig. 3) obtained. In both injection groups, the levels of Aβ and APP-CT were dramatically reduced in the cortical region of Tg-hASC group compared to the Tg-sham group (Fig. 4a). In intracerebral injection group, the levels of Aβ and APP-CT in Tg-hASC were significantly reduced (Aβ, from 1.0±0.039 to 0.55±0.018, *P*<0.05, Fig. 4b; APP-CT, from 1.0±0.033 to 0.63±0.029, *P*<0.05, Fig. 4c). In intravenous hASC injection group, Aβ and APP-CT levels in Tg-hASC mice were also significantly reduced (Aβ, from 1.0±0.039 to 0.82±0.018, *P*<0.05, Fig. 4b; APP-CT, from 1.0±0.033 to 0.78±0.029, *P*<0.05, Fig. 4c).

The level of neprilysin, one of the Aβ degrading enzymes [14], was increased in Tg-hASC mice compared to Tg-sham mice (intracerebral injection: 1.29-fold, p<0.05; intravenous injection: 1.14-fold, p<0.05; Fig. 4d), indicating increased degradation of Aβ by the induction of neprilysin.

These data provide evidence that intravenous or intracerebral hASC injections reduced not only amyloid plaque formation but also Aβ and APP-CT protein levels through the induction of neprilysin.

### Intravenous or Intracerebral hASC Transplantation Upregulated IL-10 and Neurotrophic Factors in the Brains of Tg2576

**Figure 5:**
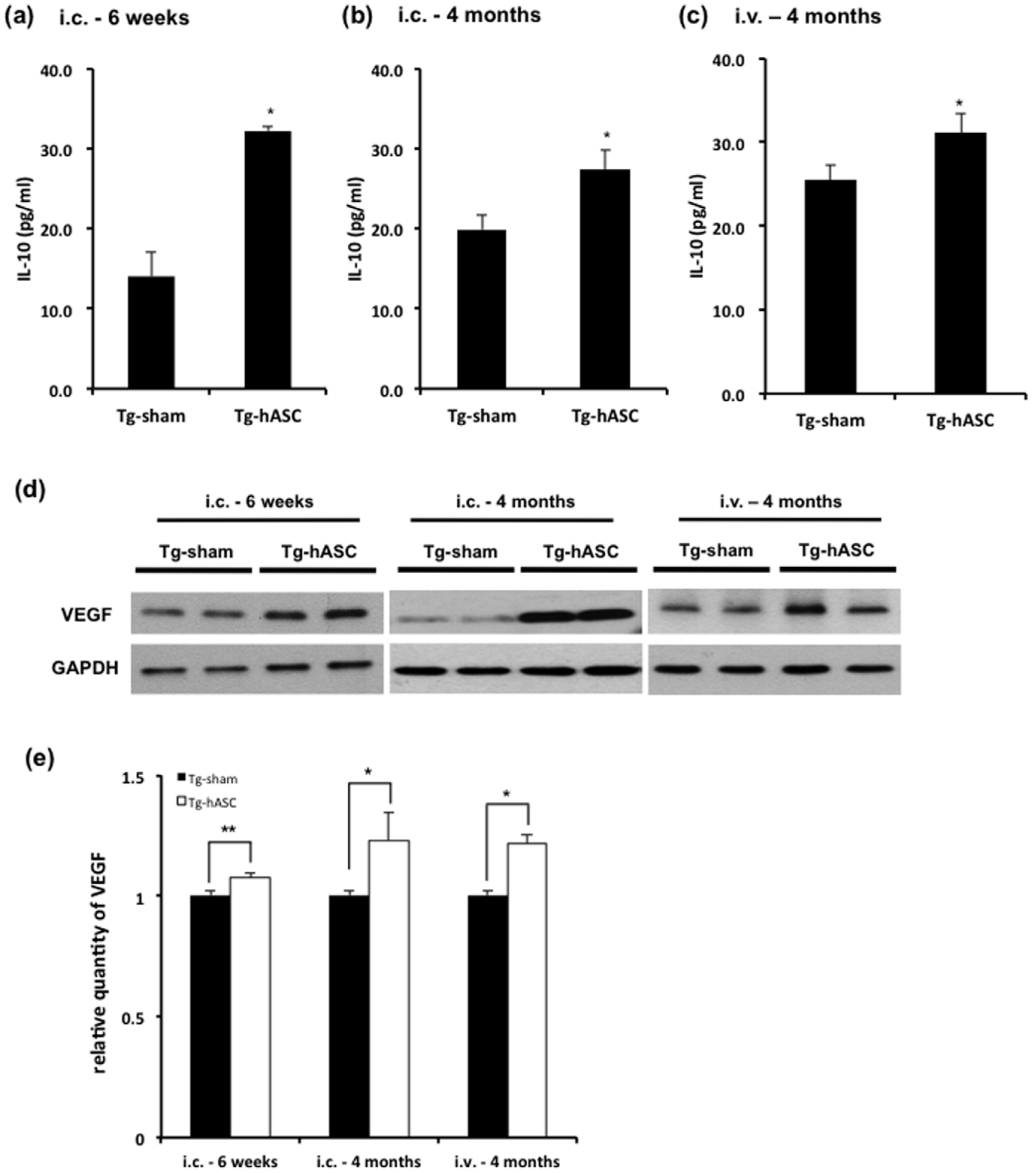
IL-10 and VEGF level were increased after intravenous and intracerebral injection of hASCs. ELISA for mouse brain lysates obtained (a) 6 weeks and (b) 4 months after the (i.c.) injection, and (c) 4 months after the 13^th^ (i.v.) injection revealed significant change in IL-10 level (n = 5). (d) Western blot of mouse brain lysates obtained from 6 weeks and 4 months after single intracerebral injection and 4 months after the 13^th^ (i.v.) injection revealed a significant increase in VEGF. (e) Quantitative data of VEGF level was obtained using western blot analysis (n = 5). All data are represented as mean ± SEM of three independent experiments. Asterisk *, *P*<0.05, **, *P*<0.01 by one-way ANOVA.

4 months after the transplantation, we found microglia were co-localized with Aβ deposition in the brains of Tg2576 mice (Fig. S2). To investigate whether transplanted hASCs mediated immune and inflammatory reaction, we quantified IL-10 and IL-1β levels by sandwich ELISA. There were significant increases in the levels of IL-10 at 6 weeks (from 14.03±3.02 to 32.16±0.58, p<0.05; Fig. 5a), 4 months (from 19.87±1.81 to 27.33±2.41, p<0.05; Fig. 5b) after intracerebral injection, 4 months after the final (13^th^) intravenous injection (from 25.47±1.78 to 31.06±2.45, p<0.05; Fig. 5c), while there was no change in IL-1β level (Fig. S2).

Next, we examined the levels of several neurotrophic factors at early and late stages after the transplantation. VEGF level also increased in the Tg-hASC group compared to the Tg-sham group at 6 weeks (from 1.0±0.02 to 1.07±0.02, p<0.01), 4 months (from 1.0±0.02 to 1.23±0.11, p<0.05) after intracerebral injection, 4 months after the final (13^th^) intravenous injection (from 1.0±0.02 to 1.22±0.03,dda p<0.05; Fig. 5e). GDNF, NT3 and NeuroD1 were significantly increased by both 3 weeks and 6 weeks after the transplantation, especially NT3 levels increased until 4 months after the injection (Fig. S3). However, there was no change in BDNF between Tg-sham and Tg-hASC groups (Fig. S3).

These results indicate that transplanted hASCs exert neuroprotective effects by inducing elevations of the anti-inflammatory cytokine IL-10 and several neurotrophic factors including VEGF, lasting for at least 4 months.

### hASC Secrete IL-10 itself, as well as Stimulate BV2 to Secrete IL-10

**Figure 6:**
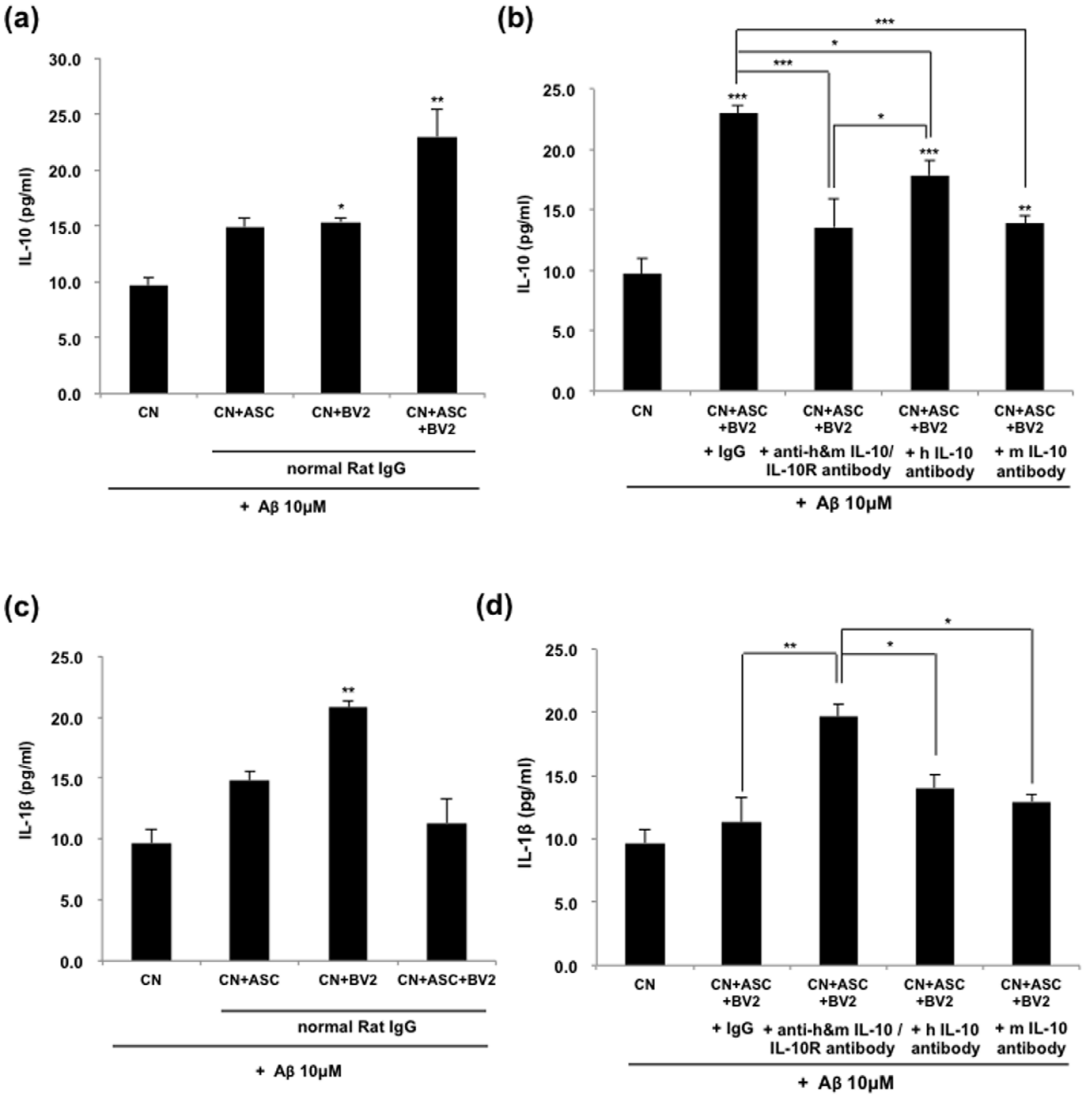
Increased IL-10 might be contributed by hASCs and the secreted level of IL-1β might be modulated by secreted IL-10 from hASCs. (a) Primary mouse neurons were grown in coated 24-well culture dishes to near confluence 80% in neurobasal media containing B27 for 7 days. They were then added to 10 µM of oligomeric Aβ_42_ peptides and co-cultured with hASCs and/or BV2 cells. Blocking of IL-10 and IL-10 receptor interaction was performed for 48 h. A neutralizing IL-10 or IL-10 receptor antibody (5 µg/ml, respectively) was used in the indicated groups and IL-10 or IL-1β ELISA was performed. (a–b) The concentration of IL-10 in hASC/BV2 co-culture system was measured with ELISA. (c–d) The concentration of IL-1β in hASC/BV2 co-culture system was measured with ELISA. Data represent mean ± SEM of three independent experiments (n = 30). Asterisk *, *P*<0.05, **, *P*<0.01, ***, *P*<0.001; by One-Way ANOVA: Tukey’s HSD Post Hoc test.

To examine which cells are responsible for the increase of IL-10 in brain tissue after hASC treatment, mouse primary neurons were co-cultured with hASC and/or BV2 cells in the presence of 10 µM oligomeric Aβ peptides, with or without IL-10 or IL-10 receptor neutralizing antibodies and was analyzed by an ELISA assay. We found that concentration of IL-10 was increased in the co-culture of hASC, BV2 or hASC/BV2 group (14.9±0.8; 15.3±0.4; 23.0±2.5 pg/ml, respectively). Especially, IL-10 was significantly increased in the co-culture of BV2 or hASC/BV2, compared to the sham group (Fig. 6a). We also investigated whether IL-10 release was blocked by treatment with antibodies for either IL-10 or IL-10 receptor. As shown in Figure 6b, IL-10 induced by co-cultured with both ASC and BV2 was significantly reduced by treatment with each antibody (13.6±1.3 pg/ml). In addition, both specific mouse IL-10 antibody (mIL-10) and specific human IL-10 (hIL-10) antibody suppressed IL-10 release induced by co-culture with hASC/BV2 (Fig. 6b). These findings suggested that hASC drove BV2 to produce IL-10 as well as hASC secretion of IL-10 itself. Moreover, hASC-medicated IL-10 production induced that neuroprotection to primary cortical neurons by a paracrine effect.

Next, we confirmed the level of IL-1β in the culture system. The levels of IL-1β were significantly increased in the primary cortical neurons co-cultured with BV2 in the presence of 10 µM oligomeric Aβ peptides (from 9.7±1.0 to 20.8±0.6 pg/ml, *p<0.05*; Fig. 6c), but IL-1β level was recovered when primary cortical neurons were co-cultured with hASC/BV2 (Fig. 6c). Treatment with blocking antibodies for either IL-10 or IL-10 receptor, the level of IL-1β significantly increased (from 11.3±1.9 to 19.6±1.0 pg/ml, *p<0.01*; Figure 6d). However, the level of IL-1β was significantly reduced in neurons treated with only one antibody of specific human or mouse IL-10 antibodies, compared with those in neurons treated with two kinds of antibody (hIL-10, 14.0±1.1 pg/ml; mIL-10, 13.5±0.5 pg/ml, *p<0.05*; Fig. 6d).

### Co-culture with hASC Significantly Reduces the Apoptotic Cell Death Induced by Oligomeric Aβ_42_

To investigate whether soluble mediators from human ASCs, especially IL-10, can directly suppress the neuronal cell death induced by oligomeric Aβ_42_, we performed TUNEL and LDH assay.

**Figure 7:**
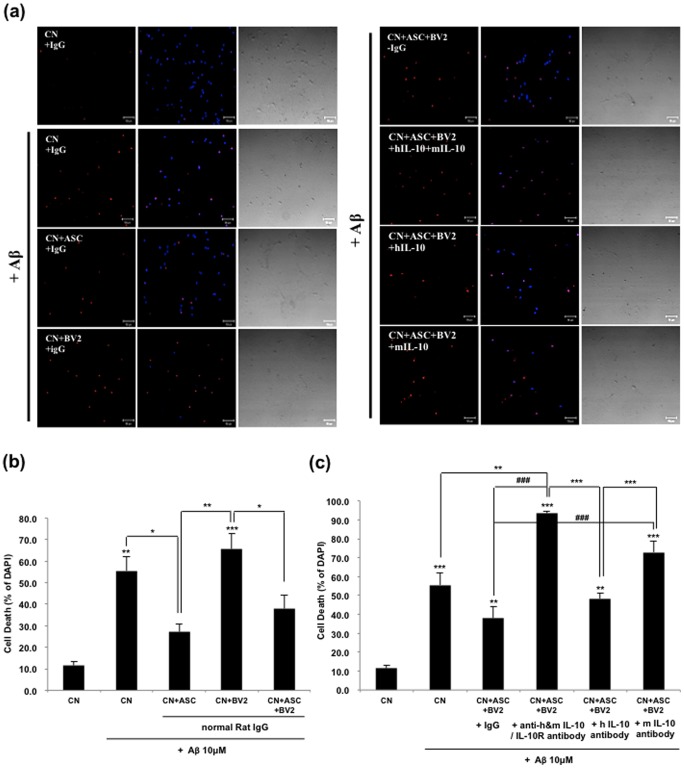
Oligomeric Aβ_42_-induced apoptotic cell death was decreased by increased IL-10 secreted from hASC and/or murine BV2 cells. (a–c) primary mouse neurons were grown in coated 24-well culture dishes to near confluence 80% in neurobasal media containing B27 for 7 days. They were then added to 10 µM of oligomeric Aβ_42_ peptides and co-cultured with hASCs and/or BV2 cells. Blocking of IL-10 receptor interaction was performed for 48 h and then LDH and TUNEL assay were performed. A neutralizing IL-10 or IL-10 receptor antibody (5 µg/ml, respectively) was used in the indicated groups. (a) Phase contrast and TUNEL staining of primary neurons treated with 10 µM of oligomeric Aβ_42_ peptides. The TUNEL-positive cells are stained red. Scale bar, 50 µm. (b, c) Data represent mean ± SEM of three independent experiments (n = 30). Asterisk *, *P*<0.05, **, *P*<0.01, ***, *P*<0.001; by One-Way ANOVA; Tukey’s HSD Post Hoc test.

We found that Aβ_42_-treated neurons displayed apoptotic nuclei and co-culture with hASC or hASC/BV2 reduced the number of apoptotic nuclei (Fig. 7a). After 48 h posttreatment of oligomeric Aβ42, the apoptotic index of cortical neurons was 55.5±6.7%, and apoptotic indexes of co-culture with hASC only, BV2 only, both hASC and BV2 were 22.7±3.2%, 65.7±7.2% and 37.96.3%, respectively, compared to the total number of cells (Fig. 7b). Neutralizing antibodies against IL-10 abrogated the neuroprotective effect of the hASCs (from 37.9±6.3 to 93.4±1.2, p<0.001; Fig. 7c).

**Figure 8:**
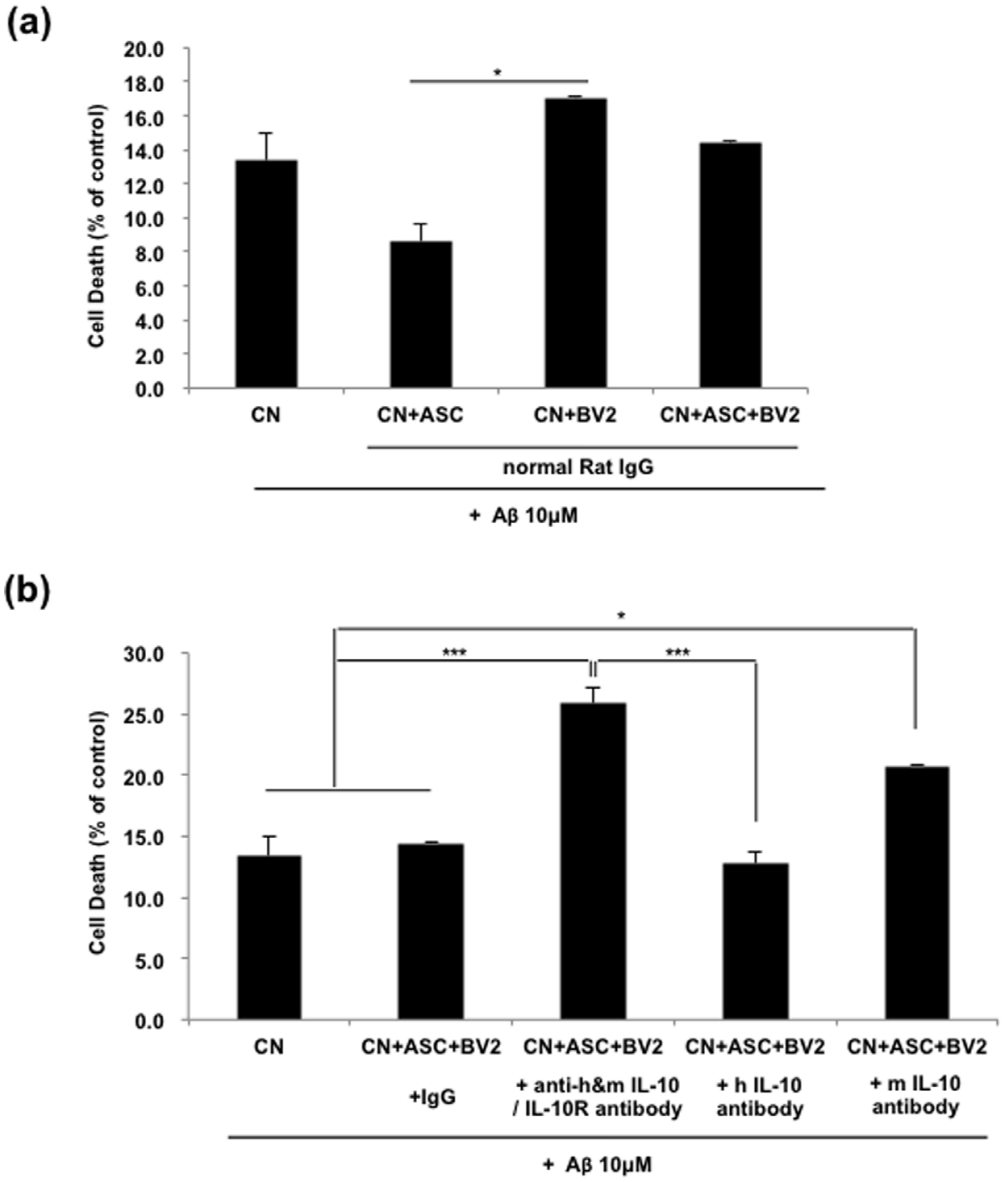
Oligomeric Aβ_42_-induced neuronal cell death was decreased by increased IL-10 secreted from hASC and/or murine BV2 cells. Primary neurons were grown in 10 µM of oligomeric Aβ_42_ with or without blocking IL-10 and IL-10 receptor interaction. After 48 h oligomeric Aβ_42_ treatment, LDH assay was performed. (a, b) The results shown are expressed as mean ± SEM from three independent experiments (n = 16). Asterisk *, *P*<0.05, **, *P*<0.01, ***, *P*<0.001; by One-Way ANOVA; Tukey’s HSD Post Hoc test.

Next, by lactate dehydrogenase (LDH) release assay, we evaluated the viabilities of mouse primary cortical neurons treated with oligomeric Aβ42. After 48 h posttreatment of Aβ42, co-culture with hASC showed significantly decreased LDH release (8.6±1.0%) versus the control (13.4±1.6%), but LDH release was increased by co-culture of BV2 (17.1±0.1%) (Fig. 8a). However, LDH release was significantly reduced to the level of the control in co-culture of both ASC and BV2 (14.4±0.1%) (Fig. 8a). Treatment with blocking antibodies for either IL-10 or IL-10 receptor significantly increased the neuronal cell death induced by oligomeric Aβ42 (from 14.4±0.1 to 25.9±1.3, p<0.001; Fig. 8b).

### Engrafted hASCs Elevated Endogenous Neurogenesis

**Figure 9:**
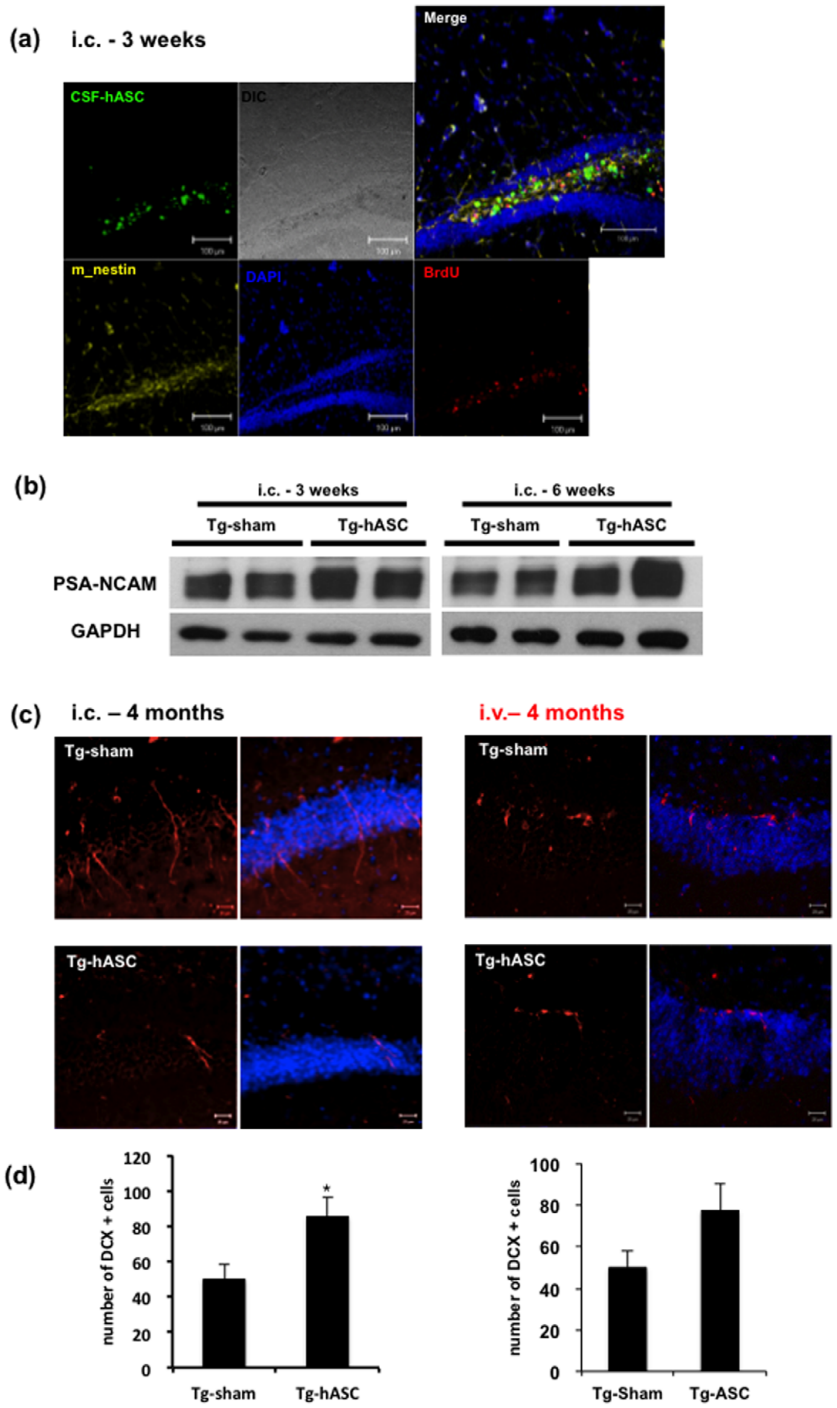
Engrafted hASCs increased endogenous neurogenesis in the brains of Tg2576 mice. (a) At 3 weeks after (i.c.) injection, the CSF-hASCs still stay at the hippocampal region with mouse Nestin and BrdU positive cells produced around them. (b) At 3 and 6 weeks after (i.c.) injection, PSA-NCAM level was increased in Tg-hASC group. One representative of three separate experiments is shown. (c-d) Immunohistochemical analysis showed significantly increased number of DCX positive cells. (c) Tissues were immunostained with anti-DCX (red) antibody and counterstained with DAPI (blue). Scale bar = 50µm. (d) Quantitative data of DCX positive cells are represented as mean ± SEM of three independent experiments (n = 8). Asterisk *, *P*<0.05 by one-way ANOVA.

Adult hippocampal neurogenesis was affected by neurotrophic or growth factors [15]. We hypothesized that hASC transplantation would induce endogenous neurogenesis through neuroprotective factors. Brain sections from mice sacrificed 3 weeks after transplantation were labeled with anti-mouse Nestin and anti-BrdU antibodies. Increased cells positive for Nestin, a primitive neurofilament protein were found around the engrafted hASCs [16] (Fig. 9a). There were some cells positive for both anti-mouse Nestin and BrdU antibodies.

To confirm the effects of hASCs on endogenous neurogenesis, we also examined neurogenesis related molecules such as PSA-NCAM. In our study, the PSA-NCAM was increased in the brains of the Tg-hASC group at both 3 and 6 weeks after the transplantation compared to the Tg-sham group (Fig. 9b). Brain sections of mice sacrificed 4 months after transplantation were labeled with anti-doublecortin (DCX) antibody and counted the DCX-positive cells in the dentate gyrus of the hippocampus (Fig. 9c). Quantitative analysis showed that endogenous neurogenesis was increased by 1.7-fold in the Tg-hASC group compared to the Tg-sham group (from 49.89±8.26 to 85.41±10.77, p<0.05; Fig. 9c).

Our immunohistochemical and western blot analyses suggest that transplanted hASCs increased endogenous neurogenesis in the hippocampus region.

### Engrafted hASCs Elevated Synaptic and Dendrite Stability

To investigate the effect of transplantation of the hASC on synaptic stability, we checked PSD-95 and synaptophysin levels in the brains of all groups. PSD-95 and synaptophysin are important factors that contribute to synaptic formation and have been proposed as a molecular scaffold for receptors and the cytoskeleton at synapses [17]. At 3 weeks and 6 weeks after the transplantation, the PSD-95 and synaptophysin were increased in the brains of hASC transplanted Tg2576 mice (Fig. S3).

**Figure 10:**
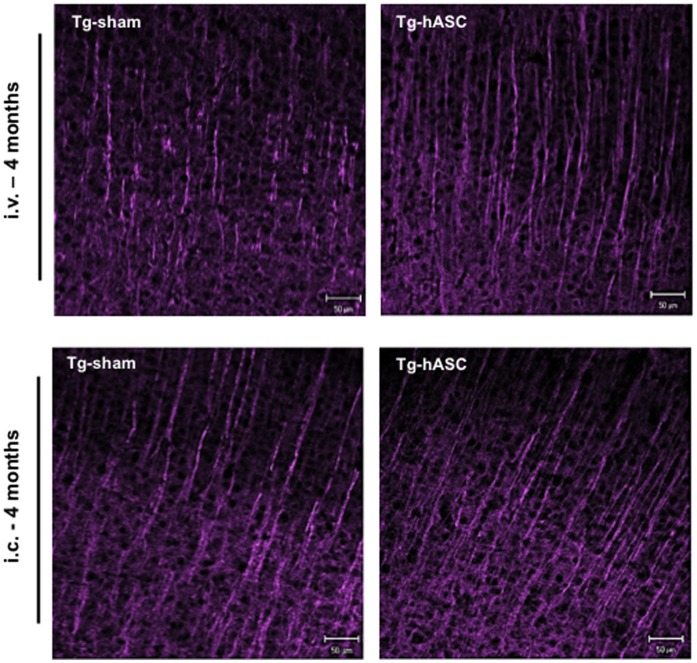
Engrafted hASCs changed morphology of neuronal dendrites in the brains of Tg2576 mice. Immunohistochemical analysis of MAP-2 showed differences between Tg-Sham and Tg-hASC group after (i.v.) and (i.c.) injection of hASC. MAP-2 stained dendrites of pyramidal cells were formed to be elongated and densely distributed in the brains of the Tg-hASC mice while MAP-2 dendrites were found to be shortened in the brains of Tg-sham mice.

Long-term changes in synaptic interaction are supposed to involve alterations in dendrite morphology [18]. Postsynaptic neuronal dendrites undergo functional and morphological changes in response to pathologically excessive synaptic activation [19]. 4 months after a single hASC injection or the final (13^th^) intravenous injection, MAP2-stained dendrites of pyramidal cells were shortened in the brains of Tg2576 mice, whereas dendrites were elongated and densely distributed in the Tg-hASC group (Fig. 10).

With the data obtained so far, we can conclude that the intravenously transplanted hASCs survive, migrate into the brain and alleviate pathology by reducing the number of amyloid plaques and memory impairment of Tg2576 mice by up-regulating IL-10 and VEGF and elevating endogenous neurogenesis and synaptic and dendritic stability.

## Discussion

There are two hallmark factors in the AD brain: amyloid plaques formed by a small peptide called Aβ and neurofibrillary tangles formed by the hyperphosphorylated microtubule-binding tau [20]. Previously, it was reported that the accumulation of Aβ plays a major role in the progression of the disease including memory impairment [21], [22]. Once progressed, pathological development is inevitable, and although there are various reports regarding a cure for AD, there is no perfect treatment that can fully diminish Aβ plaques and prevent the disease [23].

Recently, treatment of disease using stem cells has been in the spotlight due to the advantages stem cells possess. For regenerative clinical applications, stem cells should meet following criteria [24]: 1. they can be extracted in abundant quantities; 2. they can be harvested by a minimally invasive procedure; 3. they can be differentiated in to multiple cell lineages; and 4. they can be safely and effectively transplanted into either an autologous or allogenic host without immune rejection. MSCs meet the above criteria and have been shown to reduce neuronal damage and support nerve regeneration in nerve injury models [25], [26]. Among MSCs, ASCs, which originate from adipose tissue, are the most suitable for clinical application. Adipose tissue is known to contain cells that have a high proliferation capacity *in vitro* and have the ability to undergo differentiation into multiple cell lineages [27]. As adipose tissue is easily accessible, autologous ASCs transplantation like other MSCs transplantation can be performed safely without immune rejection or tumorigenesis. It has been reported that ASCs also secrete growth factors including VEGF, GDNF, NT-3, NGF and bFGF, and are thought to participate in immunization by regulating cytokine release [28], [29].

In our experiment, we showed preventive or therapeutic potential of hASCs on Tg2576 mice using two injection methods: intravenous, the simplest, convenient and safest method, and intracerebral injection. Intravenous injection is more related to preventing or delaying the onset of disease, while intracerebral injection is intended to examine the therapeutic potential of hASCs when the disease has already progressed.

Since disruption in the blood-brain barrier (BBB) was observed in the case of AD [30], after peripheral injection the amount of ASCs migrated into the brain might be increased compared with the control. We checked the organ distributions of intravenous injected hASCs using hASC labeled fluorescence magnetic nanoparticles for *in vivo* live tracking of hASCs. The present data demonstrated that fluorescent nanoparticle signals in all brain regions except the olfactory bulb (Fig. S1). Our data clearly shows that the intravenously transplanted hASCs migrate into the brain.

At first, to assess the functional effects of hASC transplantation into the AD model mouse, behavior test and pathological analysis was performed with intravenously or intracerebrally transplanted Tg or WT mice. The Morris water maze test demonstrated that spatial learning ability in the intravenously or intracerebrally transplanted Tg-hASC group was improved and this result was confirmed by the probe test. The number of amyloid plaques in Tg-hASC mice brain sections was significantly reduced compared to that of the Tg-sham group 4 months after injection. Moreover, protein levels of Aβ and APP-CT were significantly decreased in the Tg-hASC group mice brains through the induction of neprilysin till at least 4 months after injection. Other investigators checked that memory deficits and neuropathology were only reduced 2 weeks – 2 months after transplantations of stem cells [2], [8], [31].

In this study, we examined the levels of several neurotrophic or growth factors such as VEGF, GDNF, NT3, BDNF and neuroD1 (Fig. S4). Among them, VEGF, GDNF and NT3 were significantly increased by transplantation of the hASC in Tg2576 mice compared to Tg-sham group lasting for several months (Fig. S4). IL-10, anti-inflammatory cytokine was also increased in Tg-ASC mice brains for at least 4 months, while IL-1β did not change (Fig. 5). At 3 and 6 weeks post-injection, there were increased Nestin and BrdU positive cells particularly around the engrafted hASCs compared to other areas. As in previous reports, some of the injected stem cells might have sensed a damaged area, migrated, differentiated into neuronal lineages and replaced the functions of dead neurons [32]. Recent studies demonstrate an alternative mechanism referred to as the “bystander effect” of stem cell treatment [33].

Transplanted human BM-MSCs in the brains of cerebral ischemia rats induced behavioral recovery by elevating BDNF, NT-3 and VEGF levels [34], but our results showed that ASCs didn’t significantly affect the level of BDNF (Fig. S4). In addition, J. K. Lee et al. suggested that intracerebral transplantation of BM-MSCs promoted activation of microglia that secreted neurotrophic agents and resulted in cognitive improvements and reduction of Aβ pathology [8]. MSCs transplanted into the brains of cerebral infarction model rats have been reported to affect cytokine release by up-regulating IL-10 and down-regulating TNF-α [35]. Recent reports suggest that proinflammatory cytokines have a negative effect on neurogenesis, whereas anti-inflammatory cytokines exert an opposite effect [36], [37]. Growth and neurotrophic factors including VEGF has also been reported to exert neuroprotective and neurogenic effects [38].

Neuroprotective capacity of hASCs could be attributed to the soluble mediators, which was confirmed by transwell experiments. Mouse primary neurons were co-cultured with hASC or BV2 cells in the absence or presence of 10 µM oligomeric Aβ peptides, with or without IL-10 or IL-10 receptor neutralizing antibodies. BV2 activated in the presence of hASCs produced a significantly higher amount of IL-10; additionally, a blockade of IL-10/IL-10R interaction by antibodies abrogated the neuroprotective capacity of hASC culture supernatants. Therefore, we concluded that both ASC and BV2 cells affected the cell survival against Aβ by increasing IL-10. And also, we checked the secreted level of IL-1β from BV2 cells in each group and found that the level of IL-1β was decreased by co-culture with hASC. But, treatment of anti-IL-10 and anti-IL-10 receptor antibodies increased the reduced level of IL-1β again. The secreted level of IL-1β might be controlled by the secreted IL-10 from hASC. These immunomodulatory effects of hASCs might be supported by recent study, which MSCs inhibit the proliferation of either syngeneic or allogeneic T cells by inducing IL-10 [39].

Our immunohistochemical analyses suggest that transplanted hASCs increased proliferation of endogenous stem cells. We examined neurogenesis related molecules such as PSA-NCAM, which is highly expressed in the population of newly generated granule cell precursors and is closely related to neurogenesis and brain plasticity. PSA-NCAM level was also increased in the Tg-hASCs group compared to the Tg-sham group. Four months after hASC transplantation, we also found an increase of DCX positive cells, which provides additional molecular evidence for increasing the endogenous neurogenesis. These data show that the hASC transplantation is involved with the increase of endogenous neurogenesis through increasing neurotropic factors. Therefore, we suggest that intravenously or intracerebrally transplanted hASCs benefit the brain by inducing proliferation of endogenous early-stage neurons and surrounding cells in the hippocampus region. In addition, MAP2 levels were enhanced by hASC transplantation along with PSD-95, suggesting the increase of dendrite and synaptic stability.

Our previous study demonstrated that ASCs have no side effects such as tumorigenicity, chromosomal abnormalities, or immune rejection [11], confirming the systemic transplantation of hASCs in animals and humans to be safe [11]. In this study, there was also no sign of distortion or tumor formation and no noticeable anti-graft immunoreactivity.

From these data, we conclude that intracerebrally or intravenously injected hASCs dramatically improved learning and memory ability and neuropathology of Tg2576 mice by diminishing the formation of amyloid plaques, decreasing Aβ and CT levels and up-regulating IL-10, VEGF and elevating endogenous neurogenesis and synaptic and dendritic stability.

Although it is yet unclear how hASCs up-regulated IL-10 and growth factors such as VEGF and GDNF, our findings that intravenously transplanted hASCs prevent the onset and progression of the disease clearly provide an important preclinical platform for the development of prevention and therapy for AD patients.

## Materials and Methods

### Animal

All animal procedures were performed following the National Institutes of Health Guidelines for the Humane Treatment of Animals, with approval from the Institutional Animal Care and Use Committee of Seoul National University (IACUC No. SNU-091208-1). Animals of only male were used in this study.

APPswe Tg2576 mice, which express mutant human APP, Swedish (K670N/M671L) mutation, were obtained from Taconic Farms (Germantown, NY) and were bred by mating male mice with C57B16/SJL F1 females, as described by others [40]. Mice were divided into four groups (9∼15 mice per group). All mice were genotyped by polymerase chain reaction. The mice were studied at 14±0.5 months of age, which is simply described as ‘14-month-old’ or ‘14 months of age’ hereafter, except where otherwise indicated.

### Isolation and Culture of hASCs

The procedure for human Adipose-derived stem cells (hASCs) preparation was performed under GMP conditions in the Stem Cell Research Center of RNL BIO, with approval from Institutional Review Board of Seoul National University (IRB No. C-0809-009-255).

All hASCs were isolated from human adipose tissues obtained from disposed lower abdomen of patients with agreement and primarily cultured as previously described [11]. In detailed, human adipose tissues were obtained by simple liposuction from the abdominal subcutaneous fats with an informed consent and were digested with collagenase I (1 mg/mL) under gentle agitation for 60 min at 37°C. The digested tissues were filtered through a 100-µm nylon sieve, centrifuged at 470 *g* for 5 min, and then resuspended in Dulbecco’s modified Eagle’s medium (DMEM; Invitrogen)–based media containing 0.2 mM ascorbic acid and 10% fetal bovine serum (FBS). After re-centrifuging, the cell pellet was collected and cultured overnight at 37°C/5% CO_2_ in DMEM-based media containing 0.2 mM ascorbic acid and 10% FBS. The cell medium was changed to Keratinocyte-SFM (Invitrogen)-based media containing 0.2 mM ascorbic acid, 0.09 mM calcium, 5 ng/mL rEGF, and 5% FBS. The cells were maintained for 4–5 days until confluent (passage 0). When the cells reached 90% confluency, they were subculture-expanded in Keratinocyte-SFM-based media containing 0.2 mM ascorbic acid, 0.09 mM calcium, 5 ng/mL rEGF, and 5% FBS until passage 3. FBS contaminant from cultured MSCs were completely removed by several washing with PBS and was verified through the test of albumin level below the measurement limit using a bovine albumin ELISA kit (Bethyl Laboratories). The Korea Food and Drug Administration permitted the FBS-eliminated MSCs for clinical study. Aliquots of the hASCs are then tested for cell viability and fungal, bacterial, endotoxin, and mycoplasma contamination as demanded by the Code of Federal Regulations, Title 21 (21CFR) before further use. The details of specific standards are found in the 21CFR, Sections 610. No chromosomal abnormality was observed in any sample up to passage 12 [11].

### Cell Labeling with Fluorescence Magnetic Nanoparticles and Transplantation of hASCs in Tg2576 Mice

hASCs were stained with fluorescence magnetic nanoparticles commercially named CELL-STALKER™ (CSF)(BITERIALS, Seoul, Korea) at 0.2 mg/ml concentration for tracking of the intracerebral injection and with NEO-LIVE™-Magnoxide797 (BITERIALS, Korea) at 0.4 mg/ml concentration for in vivo tracking of the intravenous injection. Dissolved nanoparticles were centrifuged at 12,000 rpm for 10 minutes and the supernatant was removed. Redissolved nanoparticles with 1 ml of medium were sonicated for 5 min. After full sonication confirming the absence of floating particles, growth medium was added up to final concentration. hASCs were incubated in growth medium containing CSF or Magnoxide797 for 24 hours and washed with PBS.

For intracerebral injections, 2 µl of 1×10^5^ hASC suspension or PBS were transplanted into both hemispheres of anaesthetized 11-month old mice with Zoletil (12.5 mg/kg, VIRBAC Laboratories, Carros, France) and Rompun (17.5 mg/kg, Bayer Pharma, Puteaux, France) at 0.01 mL/g of body weight, using a Kopf stereotaxic frame (Kopf Instruments, Tujunga, CA) loaded into the hippocampus (AP, −0.15 mm; ML, ±0.13 mm; DV, −0.19 mm). hASC used in each injection came from one donor, and hASC injected at the different time period came from different donor.

For anti-BrdU staining, some mice were intraperitoneally injected with BrdU (50 mg/kg) everyday for 4 days. For intravenous injection, 150 µl of 1×10^6^ hASC suspension or PBS was injected biweekly into WT or Tg mice through the tail vein 13 times starting from the age of 3 to 10 months.

### *In Vivo* Fluorescence Imaging Study

Fluorescence images were obtained using a Maestro In Vivo Imaging System (CRi Inc., Woburn, MA, USA) for data acquisition and analysis. Before imaging, the mice were anesthetized and labeled 1×10^6^ hASCs with fluorescent nanoparticles (NEO-LIVE™-Magnoxide797, 0.4 mg/ml) were injected through tail-vein. Fluorescence measurements were performed at 5 min after the injections. For effective detection, fur on the dorsal and ventral sides of the mice was removed. Under the anesthetization, In Vivo Fluorescence measurements were performed at 1, 3, 10 and 30 days post-injection using Maestro equipment. To examine distribution of the cells, all the organs were extracted and brain regions were dissected.

In all cases, optical image sets were acquired using a deep red filter set (a band-pass filter from 671 to 705 nm and a long-pass filter from 730 to 950 nm, which were used for excitation and emission, respectively) to acquire one complete image cube. The tunable filter was automatically increased in 10-nm increments from 750 to 850 nm. A camera was used to capture images at each wavelength using a constant exposure.

### Morris Water Maze Task

The Morris water maze task was performed at 3 months after a single intracerebral injection or the last intravenous injection to measure spatial reference learning and memory based on the previously described method [41]. Three training trials per day were conducted for 6 to 7 consecutive days, with a rotation order per trial in a group. Forty-eight hours after final trial, a single probe trial was conducted. The time spent in the quadrant that previously contained the platform was recorded as the total time in the pool.

### Tissue Preparation

To obtain tissues for experiments, all animals were anaesthetized and immediately cardiac-perfused with PBS containing heparin. For morphological analyses, one hemisphere was fixed in 4% paraformaldehyde solution for 24 hours, incubated in 30% sucrose solution for 72 hours at 4°C and then sequential 25 µm coronal sections were taken on a cryostat (Cryotome, Thermo electron cooperation) and stored at 4°C. For biochemical analyses, including western blotting, enzymatic activity assays and enzyme-linked immunosorbent assay, the other half was quickly frozen on dry ice and stored at −70°C. Tissues were lysed in RIPA buffer with a cocktail of protease inhibitors (Roche).

### Antibodies

Primary antibodies were used as follows: anti-Iba1 (1∶2000, Wako), anti-6E10 (1∶1000, Covance), anti-neprilysin (1∶500, R&D systems), anti-VEGF (1∶1000, Santa cruz), anti-GDNF (1∶000, Abcam), anti-PSD-95 (1∶2000, Thermo scientific), anti-NT-3 (1∶000, Santa cruz), anti-NeuroD1 (1∶000, Millipore), anti PSA-NCAM (1∶2000, Millipore), anti-synaptophysin (1∶0000, Millipore), anti-DCX (1∶100, Santa cruz), anti- MAP2 (1∶100, Millipore), and anti-GAPDH (1∶10000, Ab frontier).

### Western Blot Analysis

Proteins were separated by SDS-PAGE and transferred to a PVDF membrane. The membrane was blocked with 5% nonfat dry milk in Tris-buffered saline, confirmed with appropriate antibodies, incubated in horseradish peroxidase-conjugated secondary antibody and detected with ECL plus solution (Amersham Pharmacia).

### Immunohistochemistry

Sections were first retrieved by 0.01 M citric acid (pH 6.0) and blocked with 0.5% triton X 100 and 2% normal serum in TBS, then incubated with primary antibody in blocking solution overnight at 4°C. For visualization under confocal microscopy, fluorescence-conjugated secondary antibodies were incubated for 1 hour at RT. Specimens were examined on Zeiss LSM 510 confocal imaging system (Zeiss, Heidelberg, Germany).

### Quantification of DCX-labeled Cells

Every 6^th^ coronal section from all animals was stained for DCX with the fluorescent immunohistochemical method using anti-DCX antibody. Positive cells were counted using a 40x objective throughout the restro-caudal extent of the granule cell layer from about 20 tissue sections. Total counted number were divided by the number of counted tissues to obtain the estimated total number of DCX-positive cells per tissue section. DCX-positive cells were calculated in mm^2^ area of dentate gyrus that was calculated from all counted tissues.

### Congo Red Staining

Hydrated sections were incubated in a freshly prepared alkaline, alcoholic, saturated sodium chloride reagent (2.5 mM NaOH in 80% reagent-grade alcohol) for 20 min at room temperature and were then incubated in 0.5% Congo red (W/V, Sigma) in an alkaline, alcoholic, saturated sodium chloride reagent (freshly prepared and filtered just prior to use) for 30 min at room temperature. Sections were washed in distilled water and counterstained with hematoxylin for 1 min. Sections were then rinsed through ascending grades of ethanol ending with 100% reagent-grade ethanol, cleared in xylene and cover slipped with permount (Fisher Scientific). With 9 to 10 brain sections of hippocampal region on one slide, we counted every plaque in the hippocampus and cortex area of each section at 200x magnification (n = 9∼15 slides).

### ELISA

ELISAs were performed using colorimetric sandwich ELISAs kits (IL-1β: Biosource International, IL-10: Invitrogen) for the quantitative determination of IL-1β and IL-10 in brains. All assays were performed according to manufacturer’s specific instructions. Levels of these proteins were calculated from a standard curve developed with specific OD versus serial dilutions of known concentration. Each standard and experimental sample was run in duplicate and the results were averaged.

### Preparation of Primary Mouse Neurons

Primary mouse neurons were derived from the cerebral cortices of the embryos (E17 days) of 6- to 7-week-old pregnant C57BL/6 mice (Japan SLC. Inc. Haruno Breeding Branch). The cerebral cortex was dissected from mouse embryo and dissociated by gentle triturate. Cells were cultured in a specified medium for neurons (neurobasal medium supplemented with B27 and penicillin-streptomycin-amphotericin B mixture [Gibco BRL]). Experiments were performed on 7-day cultures. All animal experimental procedures were performed in accordance with ‘the Guidelines of the Ethics Committee at Seoul National University’ (SNU 091208-1).

### Blocking IL-10 and IL-10 Receptor Interaction

A transwell system (1.0 µm pore size membrane, Nunc, Naperville, IL) was used to prevent hASCs or BV2 cells (mouse microglia cell line) from contacting primary neurons. Oligomeric amyloid beta (Aβ_42_) peptides were used as stimulators, and primary neurons were used as responders. 2×10^5^ primary neurons were plated on the PLL coated 12 mm round cover slip in 24 well multi-well dish. Primary neurons were loaded into the lower chamber of the well, and 1.5×10^3^ hASCs and/or BV2 cells were added to the upper chamber. Mouse primary neurons were co-cultured with hASC and/or BV2 cells in the presence of 20 µM oligomeric Aβ peptides, with or without IL-10 or IL-10 receptor neutralizing antibodies (5 µg/ml) for 48 hours.

### Cell Toxicity Assay

Using LDH assay, cell toxicity was determined at 2 days after the treatment of 10 µM oligomeric Aβ_42_. LDH activity in the medium was measured by a Cytotox 96 nonradioactive cytotoxicity assay kit (Promega) according to the manufacturer’s instructions. Absorbance was measured at 490 nm with an ELISA reader (Molecular devices, CA). The results were expressed as percentages of peak LDH release obtained on addition of vehicle (0%), and complete cell lysis following addition of 10% Triton X-100 treatment (100%).

### Evaluation of Apoptosis with TUNEL Staining

Apoptosis was assessed using the In Situ Cell Death Detection kit (Roche) according to the manufacturer’s instruction. The numbers of TUNEL (TdT-mediated X-dUTP nick end labeling)-positive cells in four random fields were quantified as an index of apoptosis, and were normalized as percentage ratios versus the total number of the cells counterstained with DAPI.

### Statistical Analysis

Data were expressed as mean ± SEM value or as ration of control value ± SEM. Statistical analysis was performed by the one-way ANOVA: Tukey’s HSD Post Hoc test using PASW statistics (SPSS version 18). The difference was considered statistically significant for Asterisk *, *P*<0.05, **, *P<*0.01, and ***, *P<*0.001.

## Supporting Information

Figure S1 Intravenously injected hASCs migrated into brain. (a) Sequential *in vivo* tracking was performed. Mice were injected with i.v. injection of LEO-Live797 labeled hASCs. Fluorescent images of the mice were taken at the indicated times. Stained hASCs were found to migrate into the brain. (b) Expression of each organ extracted 3 days after injection. (c) Expression of each brain region; OB: olfactory bulb, HP: hippocampus, CB: cerebellum, CX: cortex, MB: midbrain, BS: brain stem.
(TIFF)

Figure S2 Microglial cells were gathered around the plaques. Microglial cells, dendrites and amyloid plaques were detected by the triple staining of thioflavin S and IbaI and MAP2 antibodies.
(TIFF)

Figure S3 IL-1β level did not change after intravenous or intracerebral hASC transplantation. IL-1β level was quantified using sandwich ELISA with brain lysates obtained from mice sacrificed (a) 6 weeks and (b) 4 months after single hASCs injection. (c) IL-1β level of brain lysates obtained from groups intravenously injected with hASCs. All data represent the means ± SEM from at least five independent experiments.
(TIFF)

Figure S4 Several protein profiles in the brains of hASCs transplanted Tg2576 mice. At 3, 6 weeks and 4 months after transplantation the levels of GDNF, NT3, BDNF, NeuroD1, PSD-95 and synaptophysin were analyzed by western blot (at least three independent experiments).
(TIFF)
