# Supplementary figures and images for: The Preventive and Therapeutic Effects of Intravenous Human Adipose-Derived Stem Cells in Alzheimer’s Disease Mice
Source: PLoS One. 2012 Sep 26;7(9):e45757. doi: 10.1371/journal.pone.0045757 (PMC3458942; doi:10.1371/journal.pone.0045757)

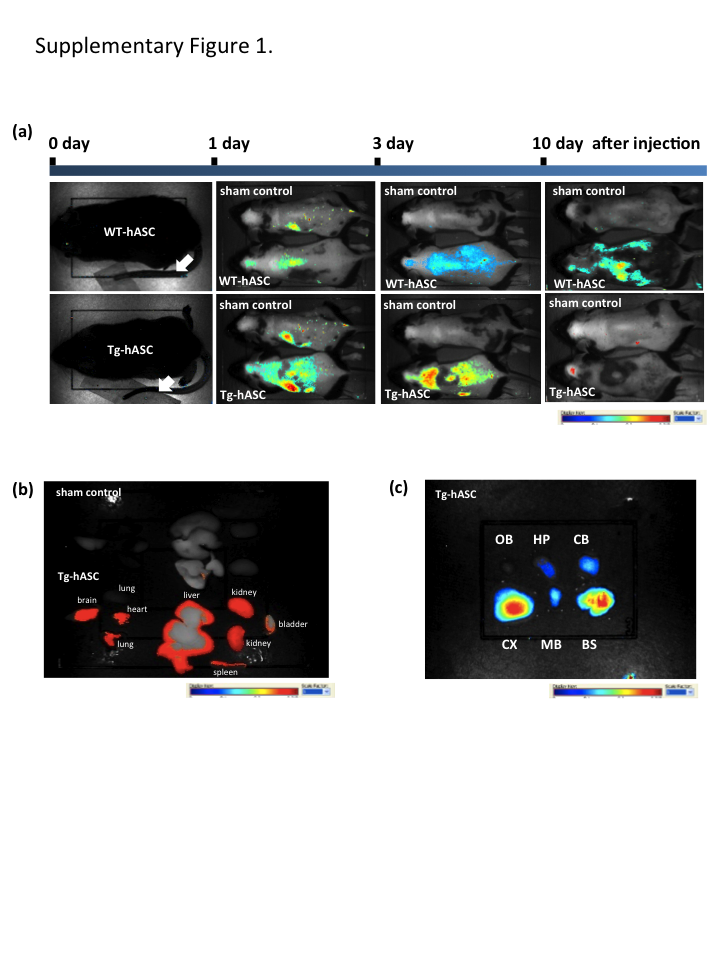

Supplement: Figure S1 — Intravenously injected hASCs migrated into brain. (a) Sequential in vivo tracking was performed. Mice were injected with i.v. injection of LEO-Live797 labeled hASCs. Fluorescent images of the mice were taken at the indicated times. Stained hASCs were found to migrate into the brain. (b) Expression of each organ extracted 3 days after injection. (c) Expression of each brain region; OB: olfactory bulb, HP: hippocampus, CB: cerebellum, CX: cortex, MB: midbrain, BS: brain stem. (TIFF) [file pone.0045757.s001.tiff]

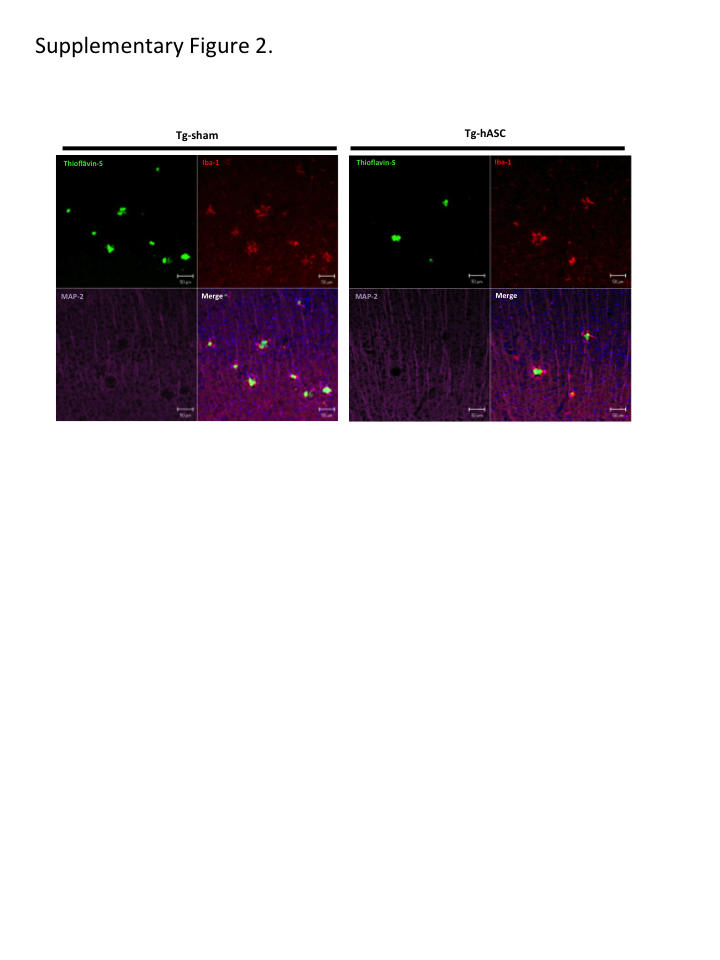

Supplement: Figure S2 — Microglial cells were gathered around the plaques. Microglial cells, dendrites and amyloid plaques were detected by the triple staining of thioflavin S and IbaI and MAP2 antibodies. (TIFF) [file pone.0045757.s002.tiff]
